# Role of Lactone and Acid Forms in the Pleiotropic Effects of Statins

**DOI:** 10.3390/pharmaceutics14091899

**Published:** 2022-09-08

**Authors:** Giulio Preta

**Affiliations:** Institute of Biochemistry, Life Science Center, Vilnius University, LT-10257 Vilnius, Lithuania; giulio.preta@bchi.vu.lt

Statins are a class of drugs used worldwide to lower low-density lipoprotein cholesterol. They are administered either as prodrug in their lactone (closed ring) or in their active hydroxy acid (open ring) form. The two forms possess significant differences in term of their physicochemical properties: the lactone form is highly lipophilic and enters the cells via passive diffusion, while the acid form has poor lipid solubility and uses active transport to enter the cells. There is evidence of a strong correlation between statin lipophilicity and pleiotropic effects, including the myopathy associated with mitochondrial complex III inhibition [1]. Beneficial pleiotropic effects of statins include anti-inflammatory, immunomodulatory and anti-proliferative properties, which are used as an adjuvant therapy in various diseases ranging from cancer therapy to Alzheimer’s, and even prevention of COVID-19 infection [2,3,4]. However, the specific contribution of the lactone and acid forms to the observed cholesterol-independent properties of statins has yet to be addressed.

The principle of the open-ring structure being the only active form of statins came into question decades ago, when Rao and colleagues reported that lovastatin lactone could modulate proteasomes activities [5]. Follow-up studies attempted to address the two forms’ contributions to the anti-cancerogenic effect of statins, with discordant results. The reason for the discrepancies is the difficulty in clearly separating the cellular effects induced by the two forms both *in vitro* and *in vivo* due to the interconversion mediated by enzymatic and pH-dependent chemical reactions [6,7]. One potential strategy for clarifying the role of the closed and open ring forms could be the utilization of alternative models, including artificial membranes, which resemble the biological membranes. For example, statins were shown to alter the nanomechanical properties of supported lipid bilayers [8], with the lactone and acid forms interacting differently with the phospholipid bilayer [9]. The lipophilicity of each statin influences how deeply they can penetrate the membrane and the intensity of the changes induced to membrane bilayers. These modifications in lipid membrane properties are biologically relevant because they are associated with changes in membrane protein functions, influencing important cellular processes via signal transduction. Moreover, variations in membrane permeability can increase drug uptake and enhance cancer cells sensitivity to chemotherapeutic agents. This novel therapeutic approach, based on the regulation of membrane lipid structure, is referred as Membrane Lipid Therapy, and is evolving rapidly due to its potential use in the treatment of several human disorders [10]. The contemporary use of both acid and lactone form of statins is an increasingly common strategy in scientific studies, and can contribute to a better understanding of the mechanisms behind statin uptake, metabolism and occurrence of pleiotropic effects [11,12] (Figure 1).

## Figures and Tables

**Figure 1:**
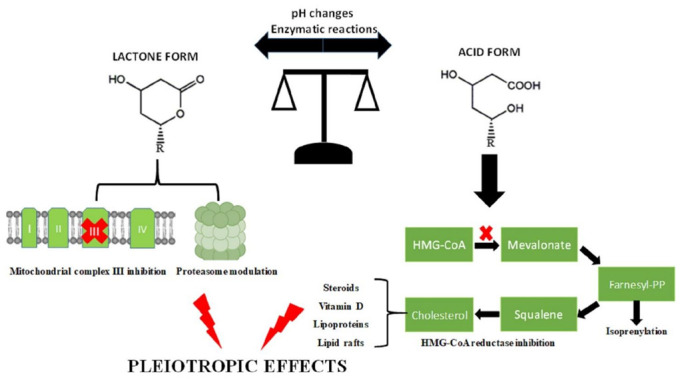
Schematic diagram for the possible mechanisms behind the pleiotropic effects of the lactone and acid forms of statins. The interconversion between the two forms is regulated by pH changes and enzymatic reactions. The lactone form inhibits mitochondrial complex III and modulates proteasome activities, while the acid form, inhibiting HMG-CoA reductase, reduces cholesterol synthesis and affects protein prenylation. Cholesterol reduction influences several other pathways, including steroids and vitamin D synthesis. Therefore, both lactone and acid forms can contribute to the pleiotropic effects of statins by different mechanisms.
